# Changes in Faecal Microbiota Profiles Associated With Performance and Birthweight of Piglets

**DOI:** 10.3389/fmicb.2020.00917

**Published:** 2020-06-11

**Authors:** Clare H. Gaukroger, Christopher J. Stewart, Sandra A. Edwards, John Walshaw, Ian P. Adams, Ilias Kyriazakis

**Affiliations:** ^1^Agriculture, School of Natural and Environmental Sciences, Newcastle University, Newcastle upon Tyne, United Kingdom; ^2^Institute of Cellular Medicine, Newcastle University, Newcastle upon Tyne, United Kingdom; ^3^Fera Science Limited, York, United Kingdom; ^4^Institute for Agri-Food Research and Innovation, Newcastle University, Newcastle upon Tyne, United Kingdom; ^5^School of Biological Sciences, Queen’s University Belfast, Belfast, United Kingdom

**Keywords:** birthweight, early-life, microbiota, performance, piglet

## Abstract

The gastrointestinal tract microbiota interacts with the host to modulate metabolic phenotype. This interaction could provide insights into why some low birthweight pigs can exhibit compensatory growth whilst others remain stunted. This study aimed to identify microbiota markers associated with birthweight [low birthweight (*n* = 13) or normal birthweight pigs (*n* = 13)] and performance [“good” or “poor” average daily gain (ADG) class]. Furthermore, the study determined whether the taxonomic markers were longitudinal, or time point specific in their ability to identify low birthweight pigs who could exhibit compensatory growth. Faecal samples were collected and liveweight recorded at 10 different time points from birth to 56 days of age. No consistent associations between birthweight, performance and gut microbiota were found across all time points. However, there was a significant (*P* < 0.05) effect of birthweight on microbiota richness at 21, 27, 32 and 56 days of age. Significant differences (*P* < 0.05) in genera abundance according to birthweight and performance were also identified. Low birthweight pigs had a significantly (*P* < 0.05) lower abundance of *Ruminococcaceae UCG-005*, but a significantly (*P* < 0.05) higher abundance of *Ruminococcaceae UCG-014* on days 21 and 32, respectively. Piglets classified as having a “good” ADG class had a significantly (*P* < 0.05) higher abundance of *Lactobacillus*, unclassified Prevotellaceae and *Ruminococcaceae UCG-005* on days 4, 8 and 14, respectively. Furthermore, *Ruminococcaceae UCG-005* was significantly more abundant at 14 days of age in normal birthweight pigs with a “good” ADG class compared to those classified as “poor.” The results of this study indicate that there are time point-specific differences in the microbiota associated with birthweight and performance, corresponding to the period in which solid feed intake first occurs. Identifying early-life microbiota markers associated with performance emphasises the importance of the neonatal phase when considering intervention strategies aimed at promoting performance.

## Introduction

The gut microbiota is now recognised for its fundamental role in moderating host health and phenotype. Increasing evidence suggests that perturbations to the neonatal microbiota development can result in a higher propensity to develop certain health disorders, including metabolic disorders and problems linked to the immune system (Schokker et al., 2014, 2015; Gensollen et al., 2016; Stiemsma and Michels, 2018). Furthermore, the neonatal microbiota can affect preterm infant growth (Grier et al., 2017) and gastrointestinal tract (GIT) physiology of piglets (Lallès et al., 2014). Thus, the neonatal period can be identified as one of the critical stages in which changes to the microbiota can have long term consequences on host health or phenotype.

As the demand for efficient pigmeat production increases, one response from the pig industry has been to increase litter size. However, this comes at the expense of average individual birthweight and litter uniformity, with a larger proportion of low birthweight (LBW) pigs born per litter (Quiniou et al., 2002; Martineau and Badouard, 2009). LBW pigs are exposed to varying degrees of intrauterine growth restriction (IUGR) (Foxcroft et al., 2006; Baxter et al., 2008; Rutherford et al., 2013), as uterine capacity to deliver nutrients to the foetuses has not increased at a rate proportionate to sow prolificacy. IUGR foetuses prioritise brain and heart development over other organs, such as the liver, GIT and the development of muscle fibres (Rehfeldt and Kuhn, 2006; Roza et al., 2008; Amdi et al., 2013). Poor development of the GIT of IUGR pigs persists pre- and post-weaning (D’Inca et al., 2011; Dong et al., 2014), with reduced rate of GIT maturation thought to negatively affect performance (Wang et al., 2005). LBW pigs represent a considerable economic problem to pig producers as a result of increased morbidity and mortality (Hales et al., 2013; Ferrari et al., 2014; Feldpausch et al., 2019), higher propensity to develop enteric health problems, poorer feed efficiency as well as carcase yield and quality with increased adiposity (Rehfeldt et al., 2008; D’Inca et al., 2011; Nissen and Oksbjerg, 2011; Zhang et al., 2018). However, a proportion of LBW pigs exhibit compensatory growth within the same environment as those who remain stunted (Paredes et al., 2012; Douglas et al., 2013; Huting et al., 2018), although an explanatory mechanism for this phenomenon is yet to be proposed.

Due to the difference in performance and health of LBW and normal birthweight (NBW) pigs and the importance of the microbiota in modulating host health, recent research has begun to explore the differences in the microbiota of LBW and NBW pigs. Early studies have shown increased bacterial adhesion in the ileum and colon of IUGR LBW pigs during early life (D’Inca et al., 2010, 2011). More recent studies have demonstrated the microbiota community composition of the jejunum, ileum, colon (Li et al., 2019; Zhang et al., 2019) and faeces (Li et al., 2018) to differ between LBW and NBW pigs up to 5 weeks of age. Whilst studies have identified microbiota characteristics related to growth of pre-weaned and weaned pigs (Mach et al., 2015; Ding et al., 2019), as well as feed efficiency of grower-finisher pigs (McCormack et al., 2017; Han et al., 2018; Quan et al., 2018), no published studies have compared the faecal microbiota of LBW piglets able to exhibit compensatory growth to those who remain stunted, in comparison to NBW pigs. Identifying early-life microbiota markers for LBW pigs able to exhibit compensatory growth could have important implications for the pig industry with regards to informing management interventions.

This study aimed to characterise the microbiota development longitudinally and capture microbiota changes associated with early life and weaning. Specific aims included identifying how the early-life microbiota development was affected by birthweight and therefore identify microbiota markers associated with piglet birthweight. Furthermore, the study aimed to identify characteristics of the microbiota associated with growth rate of LBW and NBW pigs during the pre- and post-weaning period.

## Materials and Methods

### Experimental Design and Procedures

The experiment consisted of a 2 × 2 factorial design with repeated measures taken at 10 different time points: the experimental factors were piglet birthweight (LBW or NBW) and piglet performance between birth and 56 days of age, denoted as ADG class (“poor” or “good”). Following the methodology reported by Douglas et al. (2014a), piglets from two consecutive farrowing batches were weighed, identified and classified at first handling (within the first 6–12 h of life; day 0) as being of LBW (0.80–1.25 kg) or NBW (1.50–2.00 kg). Only pigs within the LBW and NBW criteria were individually ear tagged for identification purposes (Dentag, Toptag, United Kingdom), piglets who fell outside of the LBW and NBW categories were excluded from the study. Piglet ADG was calculated between successive weighing days and for the entire experimental period (birth – 56 days of age). All piglets received a *post hoc* ADG class depending on their daily live weight gain (DLWG) for the entire experimental period compared to their birthweight (BiW) class average. Piglets within each BiW class were defined as having a “poor” ADG class if their DLWG was below the BiW class average and a “good” ADG class if their DLWG was above the BiW class average, calculated between birth – 56 days of age.

A total of 26 experimental piglets (dam line Large White x Landrace and sire line Hylean Synthetic, Hermitage Seaborough, Ltd., United Kingdom) from 10 experimental sows were used in this study. Experimental piglets formed 13 sibling pairs of LBW and NBW piglets, with three sows contributing two sibling pairs to the experimental cohort due to the antibiotic free status of their piglets. Sows were housed in farrowing crates from 110 days of gestation. Initially litter size was standardised to 12 piglets; surplus non-experimental piglets were fostered to non-experimental sows (Huting et al., 2019). No fostering of piglets into the experimental litters was permitted to reduce the introduction of foreign microbes to the pen environment. Creep feed (16.50 MJ DE/kg, 22.50% CP and 1.7% lysine; FlatDeck 1, A-One Feeds Supplements, Ltd., United Kingdom) was made available to experimental piglets from 10 days of age. None of the experimental piglets received any antibiotic treatment for the duration of the experiment.

Post-weaning, piglets were housed according to BiW class in a fully slatted, temperature-controlled weaner facility and fed a three-stage weaner diet regime. Feed and water were available *ad libitum*. Pigs received a standard three stage regime of commercial weaner diets manufactured by A-One Feed Supplements, Ltd., United Kingdom [Stage 1: 16.50 MJ DE/kg, 22.50% CP and 1.7% lysine (FlatDeck 1). Stage 2: 16.00 MJ DE/kg, 21.00% CP and 1.55% lysine (FlatDeck 150). Stage 3: 15.8 MJ DE/kg, 21.00% CP and 1.45% lysine (FlatDeck Turbowean)]. None of the diets contained antibiotics or pharmacological levels of zinc oxide. Each of the post-weaning diets was fed for 1 week to permit a period of microbiota adaption before faecal sampling occurred. This was done as a compromise between replicating commercial practice of feeding a multi-stage weaner diet and avoiding changes in diet composition from confounding the result. After the weaner diets were consumed, experimental pigs were fed a home-milled weaner meal *ad libitum* until day 56 of age (14.82 MJ DE/kg, 20.55% CP and 1.28% lysine).

Pigs were weighed and one faecal sample was collected from each experimental pig pre-weaning on days 4, 8, 14, 21 and 27 (weaning −1 day) of age. Post-weaning, pigs were again weighed and faecal sampled on days 32, 35, 42, 49 and 56 of age. All faecal samples were collected directly from the rectum during natural defecation when experimental pigs were individually isolated away from the pen and weighed. Samples were collected in 50 ml sterile plastic tubes (Sarstedt AG & Co. KG, Germany) and stored at −80°C until they were utilised for DNA extraction. A detailed description of animal housing and management can be found in the Supplementary Material S1.

### 16S rRNA Gene Profiling

Microbial genomic DNA was extracted from <250 mg of faecal sample using the DNeasy PowerSoil HTP 96 kit (Qiagen, United Kingdom) with centrifugation, following manufactures instructions. The V4 region of the 16S rRNA gene was amplified by the Nex_16S_515 F (TCGTCGGCAGCGTCAGATGTGTATAAGAGACAG**GTGYC AGCMGCCGCGGTAA**) and Nex_16S_806 R (GTCTCGT GGGCTCGGAGATGTGTATAAGAGACAG**GGACTACNVGG GTWTCTAAT**) primers (MWG, Germany). DNA was amplified by PCR (Bio-Rad C1000 Thermo Cycler, Bio-Rad Laboratories, Ltd., United Kingdom). The success of amplification was visualised using gel electrophoresis (1% agarose in TBE buffer). The amplified products were purified using Agencourt AMPure XP beads (Beckman Coulter, United Kingdom) and magnetic separation. Amplified products were then indexed using a second round of PCR (Nextera^®^ XTIndex Kit v2, Illumina, United States), then purified for a second time. The concentration of amplified products was then determined using Picogreen measured on a Fluroskan Ascent Plate Reader (Thermo Fisher Scientific, United Kingdom) and pooled in equal quantities to generate a 20 nM pool. The length of the amplified products in the pool was determined using a tapestation (2200 Tapestation, Agilent Technologies, United States) and the concentration of the pool quantified by a Qubit Fluorometer (Invitrogen, Thermo Fisher Scientific, United States). The library was then diluted to create a 4 nM pool used to generate the final amplicon library. The amplicon libraries were sequenced on the Illumina MiSeq (Illumina, San Diego, CA, United States) for paired-end fragment sizes of 300 bp.

Analysis of raw sequencing reads was performed in QIIME2 (version 2018.8, Bolyen et al., 2019). Paired-end reads were de-multiplexed and PCR primers removed (Martin, 2011). Paired-end reads were joined using “vsearch” (Rognes et al., 2016) according to the following criteria: maximum of 35 mismatches, overlap of 210 base pairs and a maximum of 260 overlapping pairs. Merged reads were quality filtered and truncated if three successive sequencing reads were below a PHRED score of 4, subsequent sequences were only retained if over 75% of the sequence length remained. Resulting merged sequences were denoised using “deblur” (Amir et al., 2017) and Amplicon Sequence Variants (referred to as OTUs) were generated. Chimeric sequences were identified using “vsearch” and removed. Taxonomic assignment to each OTU was performed by alignment to the SILVA 16S RNA database (version 132) (Quast et al., 2013). A total of 3,051,414 sequencing reads were obtained from an initial 242 piglet faecal samples run on the Illumina MiSeq (Illumina, United States). Sequencing corresponding to archaea, mitochondria and chloroplasts were removed and all sequences were rarefied to 1000 reads per sample. After rarefaction 180 samples were retained, with sample number for each piglet age group as follows: day 4 (*n* = 25), 8 (*n* = 15), 14 (*n* = 16), 21 (*n* = 20), 27 (*n* = 13), 32 (*n* = 18), 35 (*n* = 12), 42 (*n* = 19), 49 (*n* = 17) and 56 (*n* = 25). A detailed description of the sequencing analysis can be seen in Supplementary Material S2. The raw sequencing reads and associated sample metadata analysed for this study were deposited in the NCBI Sequence read Archive (SRA) under the BioProject ID: PRJNA603533 http://www.ncbi.nlm.nih.gov/bioproject/603533.

### Statistical Analysis

All statistical analyses were conducted in R version 3.4.4 (R Core Team, 2017). The main fixed effects selected for consideration from the raw experimental data in all statistical models were piglet age, BiW class and ADG class, with all possible two-way and three-way interactions between these variables being considered. Piglet ID was nested within sow ID and specified as the random effect in all models unless stated otherwise, as these variables formed the repeated measures in the dataset. Pairwise comparisons of significant fixed effects and interactions between fixed effects were determined using the “emmeans” package (Lenth et al., 2019, v 1.3.4). *P*-values were adjusted for multiple comparisons by applying the Tukey HSD method as part of the “emmeans” workflow. Resulting *P*-values below 0.05 were considered statistically significant.

All models were tested for validity, using two diagnostic plots. The first diagnostic plot consisted of a Q-Q plot of the standardised residuals, whilst the second was a scatterplot of the standardised residuals plotted against fitted values. All plots were generated by the “ggplot2” package (Wickham, 2016, v 3.1.1).

### Piglet Performance

The effect of BiW class on liveweight (kg) and ADG (kg) across all time points was tested by running a linear mixed effects model (LME) using the “nlme” package (Pinheiro et al., 2019 v3.1-140). Based on the output of the likelihood ratio Chi-squared statistic and corresponding *P*-value (*P* > 0.05) nested random effects (piglet ID nested within sow ID) did not affect the model and were removed (Zeileis and Hothorn, 2002, v 0.9-37).

### Piglet Microbiota

Observed OTUs and Shannon diversity indices were calculated using the “vegan” package (Oksanen et al., 2019, v 2.5). To determine the effects of longitudinal changes in taxonomic richness and diversity with respect to the fixed effects, a GLMER was run for observed OTUs using a Poisson regression model in the “lme4” package (Bates et al., 2015, v 1.1-21). An LME was performed with respect to the fixed effects for the Shannon indices using the “nlme” package (Pinheiro et al., 2019, v 3.1-140).

A PERMANOVA was performed using the *Adonis* function in the “vegan” package, with 999 Monte Carlo permutations, to determine whether there were longitudinal differences between microbiota community compositions based on the fixed effects. The PERMANOVA used the Bray–Curtis distance matrix (Anderson, 2001; Anderson and Walsh, 2013).

To determine how genera abundance was affected by the fixed effects of interest, relative abundance was longitudinally modelled for the top 20 most abundant genera, as these taxa were present in most pigs and time points. Taxa modelling was performed at the genera level, where taxonomic assignment permitted, by generalized linear mixed models using Template Model Builder via the “glmmTMB” package (Brooks et al., 2017, v 0.2.3) for 17 of the 20 taxa. Taxa abundance data were proportional, so within the model the family function was specified as *beta_family (link* = *“logit”)*. Model validity was assessed using the “DHARMa” package (Hartig, 2019, v 0.2.4). Models with nested random effects failed to converge for three of the 20 taxa (*Bacteroides, Escherichia–Shigella* and *Prevotella* 1) and were consequently fitted with piglet ID as a fixed variable within the model using the “betareg” package (Cribari-Neto and Zeileis, 2010, v 3.1-2). The validity of beta regression models fitted using *betareg* was determined through inspection of a half-normal plot of standardised residuals and a scatter graph of standardised residuals plotted against fitted values.

## Results

### Overview of Experimental Piglet Cohort Performance and Microbiota Composition Over the First 56 Days of Age

**FIGURE 1:**
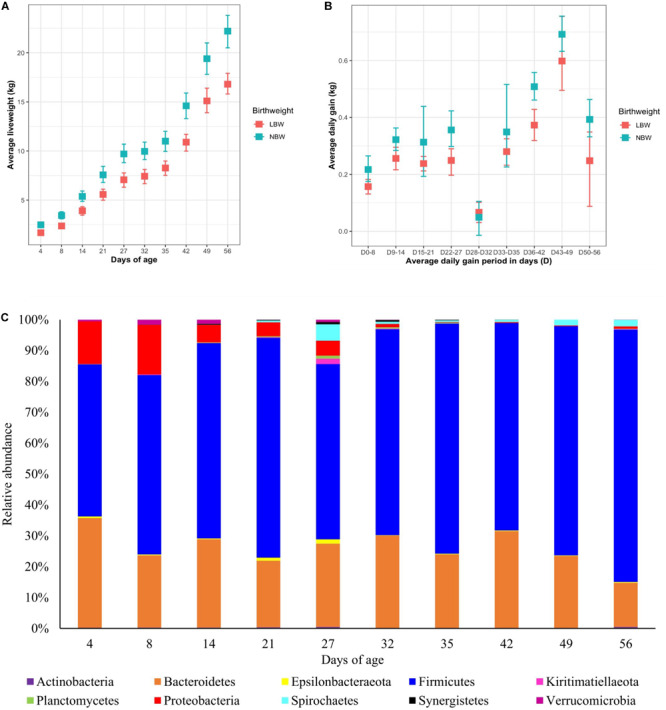
The effect of age on **(A)** mean liveweight and **(B)** average daily gain of low birthweight (LBW) and normal birthweight (NBW) pigs, with upper and lower 95% confidence intervals and on **(C)** the mean relative abundance of the 10 most abundant phyla in the faecal microbiota. Relative abundances has been rescaled to account for only the 10 most abundant phyla.

The experimental cohort displayed no symptoms of ill-health and received no antibiotics for the duration of the study. There was a significant effect of piglet age and BiW class on liveweight (*P* < 0.001), with LBW pigs consistently having a lower liveweight across all time points (Figure 1A). Whilst there was a significant effect of age on ADG, there was no significant interaction with BiW class (Figure 1B). ADG increased with age (*P* < 0.05), however a dramatic drop in ADG was associated with weaning and during the last week of the experiment, coinciding with a change in diet type with piglets transferred from commercial weaner pellets to a home-milled weaner meal. For LBW piglets the DLWG BiW class average between birth and 56 days of age was 0.28 kg/d (*SE* = 0.010), whilst for NBW piglets this was 0.37 kg/d (*SE* = 0.016).

Once all the sequences had been rarefied to 1000 reads per sample, 1700 OTUs were identified corresponding to 19 phyla, 25 classes, 41 orders, 67 families and 158 genera. At the phylum level (Figure 1C), piglet microbiota was dominated by Firmicutes and Bacteroidetes across all time points, and to a lesser extent Proteobacteria pre-weaning. Proteobacteria average abundance reduced dramatically throughout lactation, from an average of 13.4% at day 8 to 4.97% at day 27. Post-weaning average Proteobacteria abundance was less than 1% at each sampling time point. Firmicutes increased in abundance steadily between day 4 (average 49.46%) and day 56 (average 81.29%) to become the most dominant phylum. Bacteroidetes displayed less variability in abundance as piglet age increased; taxa abundance fluctuated between 4 and 56 days of age (average 35.49 and 13.82%, respectively).

The mean relative abundance of several taxa changed at critical ages in piglet management and development, such as the neonatal phase, weaning and the introduction of creep feed (Supplementary Table S1). At the genus level, *Lactobacillus* was the most dominant genus throughout the study. However, abundance sharply declined from a mean of 26.71% at 21 days of age to 4.47% at 27 days of age. Post-weaning, *Lactobacillus* abundance steadily increased to 24.68% at 56 days of age. *Bacteroides* was the second most abundant genus; abundance was highest in piglets at 4 days of age (28.82%), but steadily declined longitudinally to a mean of 0.02% in piglets at 56 days of age. *Escherichia–Shigella* was significantly abundant in neonatal piglets (10.20% at 4 days of age) but reduced as lactation progressed (2.25% at 27 days of age), post-weaning abundance declined further (0.4% average). Several initially lower abundance genera increased in mean abundance between 8 and 14 days of age, coinciding with the introduction of creep feed. These genera included: *Christensenellaceae* R-7 group (0.77% increasing to 4.49%), *Ruminococcaceae UCG-005* (0.55% increasing to 2.38%), *Rikenellaceae RC9 gut group* (0.75% increasing to 1.68%), *Prevotella 2* (0.81% increasing to 6.33%) and *Ruminococcaceae NK4A213 group* (0.75% increasing to 1.39%). Longitudinal patterns of other genera revealed an increase in mean abundance in response to weaning (27–32 days of age); these included: *Prevotella 9* (1.01% increasing to 7.65%), *Ruminococcaceae UCG-014* (1.72% increasing to 2.97%), *Subdoligranulum* (2.05% increasing to 3.19%), and *Faecalibacterium* (0.22% increasing to 2.65%).

### Associations Between Microbiota Composition and Birthweight Class

**FIGURE 2:**
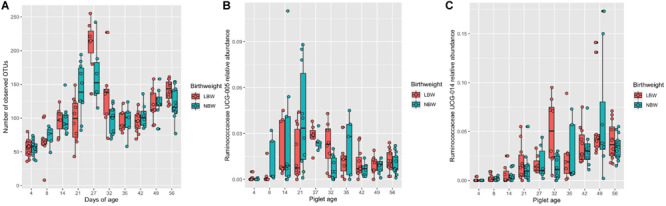
Box and whisker plots demonstrating the changes in **(A)** the number of observed OTUs, **(B)** the abundance of *Ruminococcaceae UCG-005*, and **(C)** the abundance of *Ruminococcaceae UCG-014* associated with piglet age and the interaction with birthweight class [low (LBW) or normal (NBW)]. **(A)** Significant pairwise differences in observed OTUs associated with the interaction between birthweight classes and piglet age are identified by differences in the assigned Tukey HSD letters: days 4LBW^a^, 4NBW^ab^ 8LBW^b^, 8NBW^bc^, 14LBW^cd^, 14NBW^d^, 21LBW^d^, 21NBW^g^, 27LBW^h^, 27NBW^g^, 32LBW^fg^, 32NBW^d^, 35LBW^d^, 35NBW^d^, 42LBW^d^, 42NBW^de^, 49LBW^ef^, 49NBW^ef^, 56LBW^g^ and 56NBW^f^. **(B)**. Significant differences in *Ruminococcaceae UCG-005* abundance associated with increasing piglet age and the interaction with birthweight classes (LBW or NBW) are identified by differences in the assigned Tukey HSD letters: days 4LBW^ab^, 4NWB^a^, 8LWB^abc^, 8NBW^cdef^, 14LBW^def^, 14NBW^def^, 21LBW^cd^, 21NBW^f^, 27LBW^ef^, 27NBW^def^, 32LBW^def^, 32NBW^bcd^, 35LBW^cdef^, 35NBW^cdef^, 42LBW^def^, 42NBW^def^, 49LBW^cde^, 49NBW^def^, 56NBW^def^ and 56LBW^def^ **(C)**. Significant differences in *Ruminococcaceae UCG-014* abundance associated with increasing piglet age and the interaction with birthweight classes (LBW or NBW) are identified by differences in the assigned Tukey HSD letters: days 4LBW^ab^, 4NWB^a^, 8LWB^abc^, 8NBW^abcd^, 14LBW^abcd^, 14NBW^abcd^, 21LBW^bcd^, 21NBW^cde^, 27LBW^def^, 27NBW^def^, 32LBW^f^, 32NBW^abcd^, 35LBW^def^, 35NBW^def^, 42LBW^f^, 42NBW^ef^, 49LBW^f^, 49NBW^f^, 56LBW^f^ and 56NBW^f^. The number of sample replicates for each group are presented in Supplementary Table S2.

There was a significant interaction between piglet age and BiW class for the number of observed OTUs. At 21 days of age NBW piglets had a significantly higher number of observed OTUs, whilst on days 27, 32 and 56 of age LBW pigs had a significantly higher number of OTUs (Figure 2A; in all cases *P* < 0.05). Bray–Curtis distance matrix showed no significant effect of BiW class on the overall composition of the microbiome. Modelling the top 20 most abundant genera revealed a significant effect of BiW class on the abundance of *Ruminococcaceae UCG-005* (*P* < 0.01) and *Ruminococcaceae UCG-014* (*P* < 0.01) after pairwise adjustments, with LBW pigs having a lower abundance at 21 days of age (LBW 1.16%, *SE* = 0.005 vs. NBW 3.95%, *SE* = 0.011) and higher abundance at 32 days of age (LBW 5.01%, *SE* = 0.012 vs. NBW 1.33%, *SE* = 0.004), respectively (Figures 2B,C).

### Associations Between Microbiota Composition and Average Daily Gain Class

**FIGURE 3:**
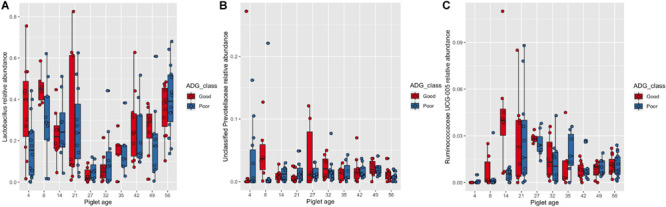
Box and whisker plot demonstrating the changes in the abundance of **(A)** *Lactobacillus*, **(B)** unclassified Prevotellaceae and **(C)** *Ruminococcaceae UCG-005* associated with piglet age and average daily gain class (Good or Poor). **(A)** Significant differences in *Lactobacillus* abundance associated with increasing piglet age and the interaction with average daily gain classes (Good or Poor). Significant differences due to the interaction are identified by differences in the assigned Tukey HSD letters: days 4Good^de^, 4Poor^abc^, 8Good^e^, 8Poor^bcde^, 14Good^abcde^, 14Poor^bcde^, 21Good^abcde^, 21Poor^bcde^, 27Good^a^, 27Poor^abcd^, 32Good^abc^, 32Poor^ab^, 35Good^abcde^, 35Poor^abcde^, 42Good^cde^, 42Poor^abcde^, 49Good^abcde^, 49Poor^abcde^, 56Good^e^ and 56Poor^e^. **(B)** Significant differences in unclassified Prevotellaceae abundance associated with increasing piglet age and the interaction with average daily gain classes (Good or Poor). Significant differences due to the interaction are identified by differences in the assigned Tukey HSD letters: days 4Good^a^, 4Poor^abc^, 8Good^c^, 8Poor^ab^, 14Good^abc^, 14Poor^abc^, 21Good^abc^, 21Poor^abc^, 27Good^abc^, 27Poor^abc^, 32Good^bc^, 32Poor^bc^, 35Good^abc^, 35Poor^abc^, 42Good^abc^, 42Poor^bc^, 49Good^abc^, 49Poor^c^, 56Good^abc^ and 56Poor^abc^. **(C)** Significant differences in *Ruminococcaceae UCG-005* abundance associated with increasing piglet age and the interaction with average daily gain classes (Good or Poor). Significant differences due to the interaction are identified by differences in the assigned Tukey HSD letters: days 4Good^a^, 4Poor^ab^, 8Good^abcd^, 8Poor^abc^, 14Good^f^, 14Poor^abcde^, 21Good^cdef^, 21Poor^cdef^, 27Good^def^, 27Poor^ef^, 32Good^cdef^, 32Poor^cde^, 35Good^abcde^, 35Poor^cdef^, 42Good^cde^, 42Poor^cde^, 49Good^bcde^, 49Poor^cdef^, 56Good^cdef^ and 56Poor^cdef^. The number of sample replicates for each group are presented in Supplementary Table S2.

There was no significant effect of ADG class on the results of the number of observed OTUs and Shannon indices, nor the Bray–Curtis distance matrix. However, the results of the most abundant 20 genera analysis showed there to be a significant association between piglet age and ADG class after pairwise adjustment. Piglets assigned to the “good” ADG class had a significantly higher abundance of *Lactobacillus* at 4 days of age (“good” 35.93%, *SE* = 0.065 vs. “poor” 16.92%, *SE* = 0.041, *P* < 0.05), unclassified Prevotellaceae at 8 days of age (“good” 4.68%, *SE* = 0.018 vs. “poor” 2.57%, *SE* = 0.024, *P* < 0.05) and *Ruminococceae UCG-005* at 14 days of age (“good” 4.25%, *SE* = 0.011 vs. “poor” 0.50%, *SE* = 0.001, *P* < 0.01) (Figures 3A–C).

### Interactive Associations Between Microbiota Composition, BiW Class and ADG Class

There were three-way interactive effects between piglet age, BiW class and ADG class on the number of observed OTUs (Supplementary Table S2). At 21 days of age, NBW piglets of both “poor” and “good” ADG class had a higher number of observed OTUs than both “poor” and “good” LBW piglets (*P* < 0.05), however on day 27 both “poor” and “good” LBW pigs had a higher number of observed OTUs compared to NBW pigs with a “good” ADG class (*P* < 0.05). At 32 days of age “poor and “good” LBW pigs had a higher number of observed OTUs compared to “poor” and “good” NBW pigs (*P* < 0.05). There was no interactive effect of BiW and ADG class on the results of the PERMANOVA analysis of the Bray–Curtis distance matrix on the overall community composition of the microbiome. However, there was a significant interaction between piglet age, BiW and ADG class, with a higher abundance of *Ruminococcaceae UCG-005* segregating NBW “good” (5.03%, *SE* = 0.032) from NBW “poor” (1.37%, *SE* = 0.008) ADG class piglets at 14 days of age (*P* < 0.001, Supplementary Table S2).

### Associations Between Microbiome Composition and Piglet Age

**FIGURE 4:**
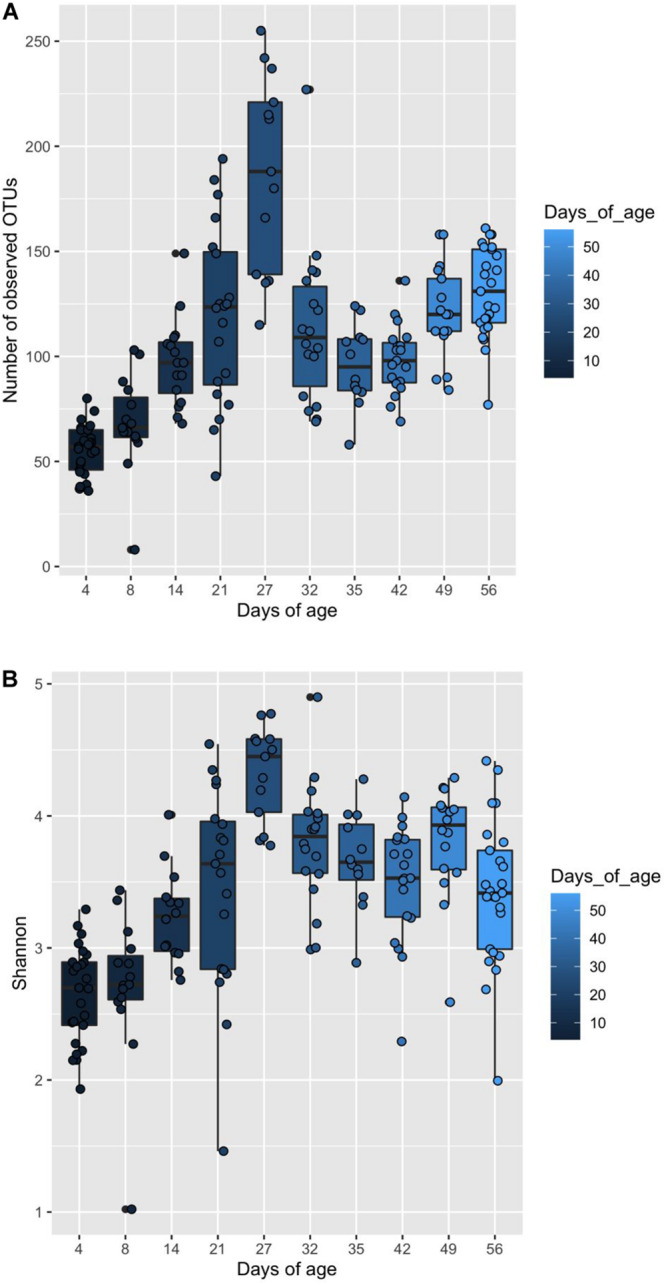
Box and whisker plot demonstrating **(A)** the changes in observed OTUs, and **(B)** Shannon diversity associated with increasing piglet age. **(A)** Significant differences in observed OTUs associated with piglet age are identified by differences in the assigned Tukey HSD letters: days 4^a^, 8^b^,14^c^, 21^d^, 27^f^, 32^d^, 35^c^, 42^c^, 49^d^ and 56^e^. **(B)** Significant differences in Shannon indices associated with piglet age are identified by differences in the assigned Tukey HSD letters: days 4^a^, 8^ab^,14^bc^, 21^cd^, 27^e^, 32^d^, 35^cd^, 42^cd^, 49^d^ and 56^cd^.

**FIGURE 5:**
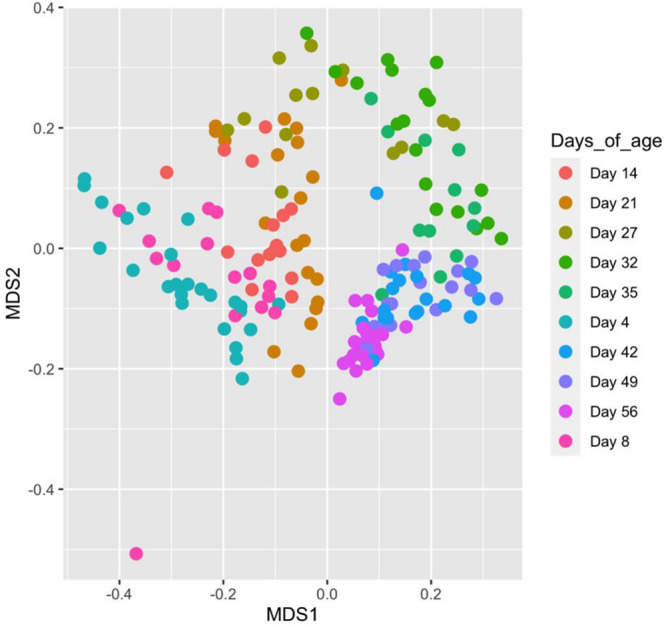
MDS plot showing changes in, and the relatedness of, faecal microbiota community composition with sample age calculated using Bray–Curtis distances. Points on the plot represent individual piglet samples which are positioned on the plot based on their similarity to other communities in two-dimensional space. Points more closely clustered represent microbiota communities more closely related to one another based on taxa composition and abundance.

**FIGURE 6:**
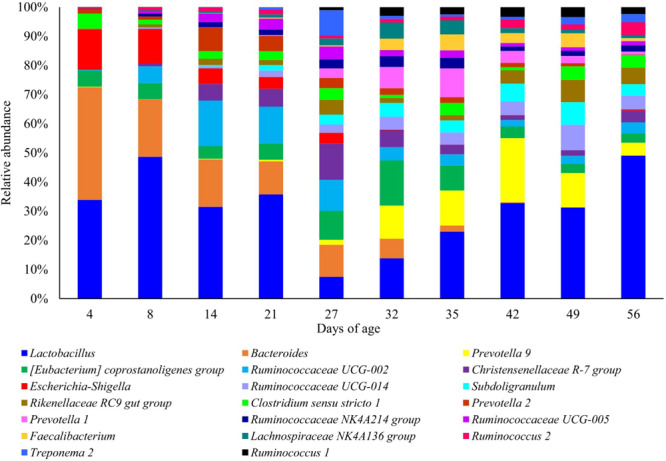
Stacked bar plot of the mean relative abundances of the 20 most abundant classified genera at each piglet age. Relative abundances were rescaled to only account for the 20 most abundant genera.

Piglet age significantly affected both the number of observed OTUs and Shannon diversity indices (*P* < 0.05) (Figures 4A,B). For both indices, alpha diversity increased exponentially until weaning where it sharply declined, followed by a steady increase and plateau in diversity. Piglet age was a significant key driver in the development of the community composition (*P* < 0.001) based on the PERMANOVA results using Bray–Curtis distances. The microbiota developed from a highly varied community composition between days 4 and 8 of age to a more uniform, stable and distinct microbiome from 35 days of age (Figure 5). All the top 20 most abundant genera were affected by age (*P* < 0.01) (Figure 6, mean raw data are presented in Supplementary Table S1).

## Discussion

The study hypothesised that microbiota markers could be identified which would be able to segregate LBW and NBW pigs, as well as pigs who exhibit superior growth rates from those who remain stunted. We found time point specific, significant associations between birthweight and the number of observed OTUs between days 21, 32 and 56 days of age. *Ruminococcaceae UCG-005* and *Ruminococcaceae UCG-014* were identified as significant taxonomic markers for BiW class at 21 and 32 days of age, respectively. Several genera were identified as age specific taxonomic markers for performance pre-weaning, whilst *Ruminococcaceae UCG-005* abundance was found to significantly differentiate NBW piglets with a superior growth rate from those piglets classified as having a “poor” ADG class. Moreover, measures of microbial diversity, community composition and genera abundance were significantly affected by age. The use of qPCR to confirm the observed significant differences in the relative abundance of the taxonomic markers associated with piglet age, BiW class and ADG class was outside the scope of this study.

In the present study LBW pigs had a consistently lower liveweight compared to NBW pigs and a lower ADG across the whole experimental period, apart from the immediate post-wean period, supporting published findings by others (Douglas et al., 2014b; Huting et al., 2017; Li et al., 2018). Whilst both NBW and LBW pigs displayed a reduction in liveweight gain associated with the post-weaning growth check (Pluske et al., 2003; Lallès et al., 2007), NBW pigs recovered faster from the growth check, with the liveweight difference between LBW and NBW pigs progressively increasing post-weaning. This pattern in liveweight, reflecting the poorer growth rates of LBW pigs, may be associated with an immature GIT (Pluske et al., 2003; Wang et al., 2010; Michiels et al., 2013), reduced creep feed intake during the lactation period (Huting et al., 2017) and consequently post-weaning anorexia or anophagia, known to induce negative effects on GIT morphology and performance (Bruininx et al., 2002; Pluske et al., 2003; Bauer et al., 2011).

The effects of piglet age and weaning on the microbiota have been widely reported in the literature and are considered as the main drivers for changes in microbiota diversity, community composition and taxa abundance (Mach et al., 2015; Bian et al., 2016; Chen et al., 2017; Han et al., 2018). Microbiota diversity increased pre-weaning, reaching a peak at 27 days of age (last day of lactation). However, this was followed by a decline in diversity immediately post-weaning, arising from weaning induced microbiota dysbiosis, before a recovery and plateau in diversity post-weaning, as reported by Frese et al. (2015) and Wang X. et al. (2019). Weaning predominantly induces community composition divergence as a result of removal of maternal milk, which is highly digestible, high in fat and contains prebiotic milk glycans; these substrates are replaced by a solid, primarily plant-based, carbohydrate rich diet (Frese et al., 2015). The uniformity of the post-weaning dietary substrates induced microbiota convergence and stability, hence the observed plateau in alpha diversity indices. The changes in beta diversity over time demonstrated neonatal community composition to display high inter-individual variability, supporting the consensus that the early life microbiota is highly stochastic and influenced by the pen and sow environment (Merrifield et al., 2016; Li et al., 2018). Overlap between the pre- and post-weaning community composition occurs at 27 days of age; this is likely to result from some piglets consuming creep feed during lactation. Whilst creep feed consumption increases between days 19 and 27 of lactation, intake is highly variable (Bruininx et al., 2002, Bruininx et al., 2004; Collins et al., 2013; Huting et al., 2017) and may also explain why measures of alpha diversity were highest at 27 days of age, with individual piglets consuming differing proportions of milk and solid feed. Irrespective of piglet birthweight and performance, the pre-weaning period was characterised by a microbiota dominated by the phyla Firmicutes, Bacteroidetes and to a lesser extent Proteobacteria. Proportions of Proteobacteria diminished as lactation progressed, while post-weaning the microbiota was dominated by Firmicutes and Bacteroidetes, as previously shown (Mach et al., 2015; Chen et al., 2017; Pollock et al., 2018). Weaning was associated with a transient reduction in certain beneficial bacteria, such as *Lactobacillus*. The microbiota shift corresponding to weaning is widely reported (Frese et al., 2015; Chen et al., 2017; Gresse et al., 2017; Valeriano et al., 2017), characterised at the family level by a reduction in Bacteroidaceae and Enterobacteriaceae, whilst Lactobacillaceae, Ruminococcaceae and Prevotellaceae increase post-weaning, arising from changes to substrate availability within the GIT (Kim et al., 2011; Pajarillo et al., 2014; Frese et al., 2015). Day 56 represented a period where experimental pigs were given a home-milled meal instead of commercial weaner pellets. The home-milled meal contained uncooked cereals altering the diet digestibility and thus fermentable substrates, giving rise to further microbiota changes.

One of the specific study aims was to establish whether there was an effect of birthweight on the microbiota profile, the age at which this occurred and the persistency of these effects longitudinally. Abnormalities in GIT physiology of IUGR LBW pigs, thought to contribute to poor mucosal immunity at birth and ADG (Wang et al., 2010; Dong et al., 2014; Hu et al., 2017; Huang et al., 2019) could influence microbiota development. Indeed, microbiota establishment in LBW human infants, particularly those born prematurely, is different to that of NBW infants (Fança-Berthon et al., 2010; Unger et al., 2015) with similar effects reported in mice (Wang et al., 2016). Differences in the microbiota community composition have been identified in LBW pigs at different intestinal sites and in the faeces (Li et al., 2018, 2019; Huang et al., 2019; Zhang et al., 2019). In the present study, differences in microbiota diversity were noted pre- and post-weaning, although inconsistent longitudinally. NBW pigs have a higher abundance of observed OTUs on day 21, but a lower abundance on days 27, 32, and 56 compared to LBW pigs, this may reflect differences in solid feed intake, particularly pre-weaning. The onset of solid feed intake characterises a key time point in microbiota development (Frese et al., 2015; Mach et al., 2015; Bian et al., 2016) and an increase in exocrine pancreatic activity and secretions, irrespective of weaning age (Pierzynowski et al., 1990, 1993, 1995). However, LBW pigs with IUGR have a reduced number of pancreatic cells, which are smaller in size and immature in neonates (Xu et al., 1994). In infants, IUGR reduces lipase activity, trypsin activity in the duodenal juice and chymotrypsin concentration in the faeces (Boehm et al., 1991; Kolacek et al., 1990). NBW pigs begin to eat creep feed sooner in lactation and consume higher volumes than LBW pigs (Huting et al., 2017), thus should have an accelerated exocrine pancreatic development compared to LBW pigs. Although creep feed intake was not directly measured in this study, a presumed higher feed intake amongst NBW pigs, possible alterations to digestive secretions and consequently composition of fermentable substrates, is the mostly likely explanation for a higher number of OTUs present in NBW pigs on day 21 of the experiment. To validate this assumption, future studies should monitor daily creep feed intake of both LBW and NBW pigs. Poor intestinal maturation persists post-weaning, with LBW pigs slower to adapt to solid feed, indicated by the reduced thickness of the *tela mucosa* and *tunica muscularis* (Michiels et al., 2013) and reduced proximal aminopeptidase A and maltase, although Huygelen et al. (2015) did not report a difference in brush border enzyme activity between LBW and NWB pigs. LBW pigs have also been shown to exhibit a different caecum fermentation pattern with a lower pH and increased concentrations of acetate and propionate compared to NBW pigs (Michiels et al., 2013). Delayed creep feed intake, slower GIT maturation and altered fermentation patterns may cumulatively explain the significant increase in microbiota diversity on days 27, 32 and 56 arising from changes to the GIT environment and substrate availability.

No differences were observed between LBW and NBW pigs longitudinally regarding community composition (beta diversity), supporting findings reported for NBW and LBW infants, mice and pigs (Costello et al., 2013; Wang et al., 2016; Li et al., 2019). The present study found age specific differences in taxa abundance between LBW and NBW pigs. *Ruminococcaceae UCG-005* was higher in abundance pre-weaning for NBW pigs. Zhang et al. (2019) also reported abundance to be higher in the jejunum of NBW pigs at day 21 of age, although Li et al. (2018) reported the abundance of *Ruminococcaceae UCG-005* to be higher in LBW pigs on days 7 and 21 of age. Both the current study and Li et al. (2018) report *Ruminococcaceae UCG-005* and *-014* to be within the top 50 taxa of both LBW and NBW pigs and faecal abundance to increase with age. These bacteria ferment dietary fibre and produce short chain fatty acids (SCFAs) (Song et al., 2017; Liu et al., 2018), and are considered stable microbiota component of the caecum and colon, irrespective of BiW class. As with these results on faecal microbiota, inconsistent findings were reported by studies comparing the ileal microbiota of LBW and NBW pigs (Li et al., 2019; Zhang et al., 2019). Differences in the results may arise from factors related to BiW class criteria, genetics, weaning age and management procedures (including age at creep feed introduction), amplicon library preparation and data analysis. In the present study, the LBW pigs were heavier at birth compared to other studies which describe IUGR to modulate LBW pig physiology, induce GIT immaturity and compromise mucosal immunity (D’Inca et al., 2010, 2011; Michiels et al., 2013; Dong et al., 2014; Li et al., 2018, 2019; Zhang et al., 2019). This may explain the reported minor effects of BiW class on the microbiota in the present study.

To date, only limited research has been conducted to explore how characteristics of the early life microbiota are associated with piglet performance (Mach et al., 2015; Han et al., 2018; Ding et al., 2019); therefore an additional aim of the study. In the present study, there was an interaction between BiW class and ADG class over specific time points for observed OTUs, corresponding to late lactation and the immediate post-weaning period, although the results were not consistent over time. However, similar microbiota diversity fluctuations over time between pre-ruminant calves of high and low growth rates have also been reported (Oikonomou et al., 2013). In addition, a study comparing the faecal microbiota of LBW and NBW mice able to exhibit compensatory growth reported that LBW mice exhibit microbiota dysbiosis during early life (Wang et al., 2016). Mach et al. (2015) identified enterotype-like clusters associated with performance of piglets, with increased pre-weaning performance associated with piglets classified within the Ruminococcaceae enterotype. In this study we identified *Ruminococcaceae UCG-005* abundance to be positively associated with performance pre-weaning; moreover, a significant interaction with BiW classes was observed, with a 3.66% higher abundance in NBW “good” pigs than NBW “poor” pigs at 14 days of age. The Ruminococcaceae family is specialised to degrade and ferment dietary fibre containing complex polysaccharides (Biddle et al., 2013). A by-product of this fermentation is the production of SCFAs, with *Ruminococcaceae UCG-005* abundance positively correlated with SCFA concentration in the faeces of piglets (Liu et al., 2018; Zhu et al., 2020). SCFAs can be utilised by the piglet as an energy source to support growth. A higher abundance of this taxa could therefore be a marker of dietary fibre and thus solid feed intake. Presence of this genera pre-weaning will help to prime the gut to utilise plant-based carbohydrates and may help the GIT to adapt to dietary substrate changes experienced during weaning. A moderate increase in the dietary fibre content of creep feed has shown to alter the microbiota composition in pre-weaned piglets (Zhang et al., 2016), although not specifically *Ruminococcaceae UCG-005* abundance. Further research exploring the specific effect of different fibre sources on *Ruminococcaceae UCG-005* abundance is required.

Unclassified Prevotellaceae was significantly higher in pigs with a “good” ADG class on day 8 only. The Prevotellaceae family is able to degrade complex carbohydrates in plant-based feeds, with abundance correlated to fibre content of the feed (Le Sciellour et al., 2018). Whilst creep feed was not introduced until day 10, it was not possible to prevent piglets from eating spilled sow feed. Higher abundance of unclassified Prevotellaceae associated with “good” pigs could therefore also be an indication of early consumption of solid feed, however it could also arise from these piglets consuming larger quantities of milk. Unclassified Prevotellaceae abundance has been shown to be correlated with lactose concentration of milk (Bian et al., 2016), so a higher total intake of lactose may induce the same effect. *Lactobacillus* was also significantly higher in abundance in “good” ADG class piglets at 4 days of age and only numerically higher at 8 days of age; Ding et al. (2019) similarly reported *Lactobacillus* abundance to be positively correlated with performance pre-weaning. Lactobacilli have a beneficial effect on weight gain of very low birth weight infants when provided as a probiotic during early life (Vendt et al., 2006; Härtel et al., 2017) as well as in piglets pre- and post-weaning (Liu et al., 2015; Wang Y. et al., 2019). This effect may be mediated by increasing production of lactic acid, thus altering GIT pH, inducing a beneficial shift in microbiota taxa abundance or establishment and reducing the degree of inflammatory response (Kim et al., 2007; Liu et al., 2014; Hou et al., 2015). Therefore, one option to increase *Lactobacillus* abundance in early life is to administer strains of Lactobacilli orally to neonatal piglets (Yang et al., 2020). Aligning probiotic administration with routine neonatal practices, such as teeth clipping, will help to limit handling stress whilst implementing this potential intervention strategy.

## Conclusion

The study is the first to compare piglet birthweight and its interaction with performance in relation to longitudinal characteristics of the microbiota in early life. The main findings support the premise that timing of solid feed intake modulates the significant age-related microbiota shifts. Time point specific microbiota markers associated with ADG and BiW classes could be identified. Future studies, with greater replication, should not only continue to define the microbiota of viable LBW pigs that exhibit compensatory growth but focus research to understand the factors which affect microbiota establishment during early life. However, early life appears to be the critical period in which modulations to the microbiota can induce significant beneficial effects on long term performance of both LBW and NBW pigs.

## Data Availability Statement

The datasets generated for this study can be found in the NCBI Sequence read Archive (SRA) BioProject ID: PRJNA603533.

## Ethics Statement

Animal experimental procedures were conducted between January and March 2017 at the Pig Research Centre at Cockle Park Farm, Newcastle University (Ulgham, Morpeth, United Kingdom). All experimental procedures were conducted after approval from the Animal Welfare and Ethics Review Board (AWERB) of Newcastle University, Newcastle upon Tyne, United Kingdom (AWERB project ID no. 555). Pig management and housing adhered to UK legislation and were in accordance with commercial standards specified by the Red Tractor assurance scheme (Red Tractor Assurance, 2019).

## Author Contributions

IK and SE conceived the research. CG, IK, and SE designed the experiment. CG conducted the experiment. CG and IA performed the analysis of the samples. CG, JW, IK, and CS analysed the data. All authors contributed to the manuscript development and interpretation and read and approved the final manuscript.

## Conflict of Interest

IA and JW are employed by Fera Science, Ltd. The funder Fera Science, Ltd. had the following involvement with the study: 16S metagenomic sequencing library preparation, analysis, interpretation of data, decision to publish and preparation of the manuscript, via authors IA and JW.

The remaining authors declare that the research was conducted in the absence of any commercial or financial relationships that could be construed as a potential conflict of interest.

## Abbreviations

ADG class: average daily gain class
BiW class: birthweight class
bp: base pairs
CFUs: colony forming units
DLWG: daily liveweight gain
GIT: gastrointestinal tract
IUGR: intrauterine growth restriction
LBW: low birthweight
lme: linear mixed effect
NBW: normal birthweight
OTU: operational taxonomic unit
PCR: polymerase chain reaction
PERMANOVA: permutational multivariate analysis of variance
SCFAs: short chain fatty acids
TBE: *tris*-borate-EDTA.
