# Supplementary material for: Changes in Faecal Microbiota Profiles Associated With Performance and Birthweight of Piglets
Source: Front Microbiol. 2020 Jun 11;11:917. doi: 10.3389/fmicb.2020.00917 (PMC7300224; doi:10.3389/fmicb.2020.00917)
Supplement: Supplementary file 1 [file Table_1.docx]

**Supplementary Table S1**

**Supplementary Table S1**. Changes in the relative abundance of the top 20 genera over time. All *P* values were FDR adjusted for multiple comparisons (Benjamini and Hochberg, 1995^†^), *P* values below 0.05 were considered statistically significant. Significant differences in genera abundance between days of age are identified by differences in the assigned Tukey HSD letters.

| Genera | Days of age | | | | | | | | | | FDR adjusted *P* values |
| --- | --- | --- | --- | --- | --- | --- | --- | --- | --- | --- | --- |
|  | 4 | 8 | 14 | 21 | 27 | 32 | 35 | 42 | 49 | 56 |  |
| Unclassified Prevotellaceae | 0.0285^a^ | 0.0342^ab^ | 0.0109^ab^ | 0.0116^ab^ | 0.0262^ab^ | 0.0207^b^ | 0.0148^ab^ | 0.0179^b^ | 0.0202^b^ | 0.0094^ab^ | 1.05E-04 |
| *Prevotella 2* | 0.0115^a^ | 0.0081^ab^ | 0.0633^c^ | 0.0396^c^ | 0.0213^c^ | 0.0157^bc^ | 0.0129^bc^ | 0.0113^bc^ | 0.0076^bc^ | 0.0014^ab^ | 3.05E-11 |
| *Prevotella 9* | 0.0012^a^ | 0.0002^a^ | 0.0018^a^ | 0.0047^ab^ | 0.0101^a^ | 0.0765^bc^ | 0.0784^cde^ | 0.1657^e^ | 0.0838de | 0.0344^cd^ | 1.99E-42 |
| *Rikenellaceae RC9 gut group* | 0.0025^a^ | 0.0075^ab^ | 0.0168^bc^ | 0.0131^bc^ | 0.0310^cd^ | 0.0119^abc^ | 0.0117^bc^ | 0.0341^de^ | 0.0548^e^ | 0.0432^de^ | 1.94E-38 |
| *Lactobacillus* | 0.2529^a^ | 0.3592^cd^ | 0.2468^cd^ | 0.2671^cd^ | 0.0447^a^ | 0.0924^ab^ | 0.1495^abc^ | 0.2468^cd^ | 0.2226^bcd^ | 0.37824^d^ | 1.64E-11 |
| *Christensenellaceae R-7 group* | 0.0006^a^ | 0.0077^ab^ | 0.0449^de^ | 0.0459^cd^ | 0.0747^e^ | 0.0388^bcd^ | 0.0213^ab^ | 0.0114^abc^ | 0.0139^bcd^ | 0.0310^d^ | 5.09E-22 |
| *Clostridium sensu stricto 1* | 0.0382^c^ | 0.0123^abc^ | 0.0204^c^ | 0.0222^bc^ | 0.0241^c^ | 0.0064^ab^ | 0.0274^abc^ | 0.0079^a^ | 0.0328^abc^ | 0.0337c | 1.67E-06 |
| Unclassified Lachnospiraceae | 0.0267^ab^ | 0.0197^a^ | 0.0288^abc^ | 0.05995^cde^ | 0.0605^de^ | 0.0750^de^ | 0.1249^f^ | 0.0709^de^ | 0.0855^ef^ | 0.0453^bcd^ | 9.71E-25 |
| *Eubacterium [coprostanoligenes] group* | 0.0412^a^ | 0.0413^ab^ | 0.0341^a^ | 0.0419^ab^ | 0.0601^ab^ | 0.1035^b^ | 0.0560^ab^ | 0.0297^a^ | 0.0221a | 0.0242^a^ | 2.16E-06 |
| *Faecalibacterium* | 0.0001^a^ | 0.0001^a^ | 0.0008^a^ | 0.0028^ab^ | 0.0022^ab^ | 0.0265^bc^ | 0.0354^d^ | 0.0256^cd^ | 0.0338^d^ | 0.0077^bc^ | 2.08E-30 |
| Unclassified Ruminococcaceae | 0.0019^a^ | 0.0060^ab^ | 0.0138^bcd^ | 0.0105^bc^ | 0.0170^cde^ | 0.0151^cde^ | 0.0263^cde^ | 0.0185^cde^ | 0.0262^e^ | 0.0209^de^ | 3.18E-23 |
| *Ruminococcaceae NK4A214 group* | 0.0010^a^ | 0.0075^ab^ | 0.0139^bc^ | 0.0143^c^ | 0.0189^c^ | 0.0247^bc^ | 0.0238^bc^ | 0.0116^bc^ | 0.0127^bc^ | 0.0170^bc^ | 5.17E-15 |
| *Ruminococcaceae UCG-002* | 0.0025^a^ | 0.0424^bc^ | 0.1226^e^ | 0.0942^de^ | 0.0645^cde^ | 0.0308^b^ | 0.0251^bcd^ | 0.0171^b^ | 0.0200^bc^ | 0.0296^bc^ | 3.47E-30 |
| *Ruminococcaceae UCG-005* | 0.0003^a^ | 0.0055^ab^ | 0.0238^cd^ | 0.0279^cd^ | 0.0267^d^ | 0.0141^c^ | 0.0168^bc^ | 0.0089^c^ | 0.0088^c^ | 0.0109^cd^ | 1.15E-19 |
| *Ruminococcaceae UCG-014* | 0.0002^a^ | 0.0021^ab^ | 0.0058^ab^ | 0.0162^bc^ | 0.0172^cd^ | 0.0297^c^ | 0.0273^cd^ | 0.0345^de^ | 0.0620^e^ | 0.0368^de^ | 1.99E-42 |
| *Ruminococcus 2* | 0.0036^a^ | 0.0083^ab^ | 0.0109^abc^ | 0.0119^a^ | 0.0055^ab^ | 0.0085^ab^ | 0.0078^ab^ | 0.0220^cd^ | 0.0125^bcd^ | 0.03404^d^ | 5.22E-19 |
| *Subdoligranulum* | 0.0000^a^ | 0.0014^ab^ | 0.0033^ab^ | 0.0143^bc^ | 0.0205^cd^ | 0.0319^de^ | 0.0269^def^ | 0.0474^ef^ | 0.0552^f^ | 0.0300^def^ | 2.86E-50 |
| *Bacteroides* | 0.2882^e^ | 0.1456^d^ | 0.1276^d^ | 0.0847^cd^ | 0.0672^bcd^ | 0.0451^bc^ | 0.0136^ab^ | 0.0019^a^ | 0.0002^a^ | 0.0002^a^ | 9.79E-14 |
| *Escherichia-Shigella* | 0.1020^e^ | 0.0871^de^ | 0.0417^cd^ | 0.0305^bc^ | 0.0225^ab^ | 0.0014^a^ | 0.0003^a^ | 0.0008^a^ | 0.0001^a^ | 0.0033^a^ | 1.02E-10 |
| *Prevotella 1* | 0.0002^a^ | 0.0001^a^ | 0.0006^ab^ | 0.0019^ab^ | 0.0197^ab^ | 0.0488^d^ | 0.0604^d^ | 0.0306^cd^ | 0.0178^bc^ | 0.0078^ab^ | 3.33E-16 |

† Benjamini, Y., and Hochberg, Y. (1995). Controlling the False Discovery Rate: A Practical and Powerful Approach to Multiple Testing. *J. R. Stat. Soc. Series. B.* 57 (1), 289-300. doi: 10.1111/j.2517-6161.1995.tb02031.x
