# Supplementary material for: Changes in Faecal Microbiota Profiles Associated With Performance and Birthweight of Piglets
Source: Front Microbiol. 2020 Jun 11;11:917. doi: 10.3389/fmicb.2020.00917 (PMC7300224; doi:10.3389/fmicb.2020.00917)
Supplement: Supplementary file 2 [file Table_2.docx]

**Supplementary Table S2**

| Days of age * BiW class * ADG class | Number of observed OTUs | *Ruminococcaceae UCG-005* relative abundance | Replicates (n =) |
| --- | --- | --- | --- |
| 4 LBW Good | 51^a^ | 1.00E-06^a^ | 6 |
| 4 LBW Poor | 60^abcd^ | 0.0009^abcde^ | 5 |
| 4 NBW Good | 59^abc^ | 1.00E-06^ab^ | 4 |
| 4 NBW Poor | 54^ab^ | 0.0001^ac^ | 4 |
| 8 LBW Good | 65^abcd^ | 0.0045^abcdef^ | 4 |
| 8 LBW Poor | 67^bcde^ | 0.0003^abcd^ | 6 |
| 8 NBW Good | 77^abcdefg^ | 0.0135^bcdefghi^ | 5 |
| 8 NBW Poor | 70^abcdef^ | 0.0117^bcdefghi^ | 7 |
| 14 LBW Good | 107^fghijknpq^ | 0.0270^fghi^ | 5 |
| 14 LBW Poor | 70^bcdefgh^ | 0.0060^abcdefghi^ | 2 |
| 14 NBW Good | 99^efghijk^ | 0.0683^i^ | 3 |
| 14 NBW Poor | 98^ghijklm^ | 0.0047^abcdef^ | 6 |
| 21 LBW Good | 98^fghijk^ | 0.0136^abcdefgh^ | 5 |
| 21 LBW Poor | 96^fghijklmno^ | 0.0190^abcdefgh^ | 5 |
| 21 NBW Good | 163^uv^ | 0.0450^hi^ | 4 |
| 21 NBW Poor | 127^pqrstu^ | 0.0358^fghi^ | 6 |
| 27 LBW Good | 201^w^ | 0.0294^ghi^ | 5 |
| 27 LBW Poor | 226^w^ | 0.0260^fghi^ | 2 |
| 27 NBW Good | 139^ijklmnopqrstuv^ | 0.0250^cdefghi^ | 1 |
| 27 NBW Poor | 169^vw^ | 0.0246^ghi^ | 5 |
| 32 LBW Good | 136^orstu^ | 0.0240^fghi^ | 4 |
| 32 LBW Poor | 132^qstu^ | 0.0210^fghi^ | 4 |
| 32 NBW Good | 101^efghi^ | 0.0080^abcdefgh^ | 4 |
| 32 NBW Poor | 96^fghikl^ | 0.0070^abcdef^ | 6 |
| 35 LBW Good | 83^defghi^ | 0.0053^abcdefg^ | 3 |
| 35 LBW Poor | 105^hijklmnopqrs^ | 0.0183^fghi^ | 4 |
| 35 NBW Good | 91^cdefgh^ | 0.0240^bcdefghi^ | 2 |
| 35 NBW Poor | 99^ghijklmnopqrs^ | 0.0213^bcdefghi^ | 3 |
| 42 LBW Good | 90^efghi^ | 0.0075^abcdefgh^ | 6 |
| 42 LBW Poor | 102^ghijklmnopr^ | 0.0142^fghi^ | 5 |
| 42 NBW Good | 107^hijklmnopqrs^ | 0.0083^bcdefghi^ | 4 |
| 42 NBW Poor | 99^fghijklm^ | 0.0050^abcdefgh^ | 4 |
| 49 LBW Good | 132^lmorstu^ | 0.0088^abcdefgh^ | 5 |
| 49 LBW Poor | 103^hijklmnopqrs^ | 0.0090^bcdefghi^ | 4 |
| 49 LBW Good | 116^hijklmnopqrst^ | 0.0070^bcdefghi^ | 3 |
| 49 LBW Poor | 128^nopqrstu^ | 0.0096^efghi^ | 5 |
| 56 LBW Good | 140^stuv^ | 0.0106^defghi^ | 5 |
| 56 LBW Poor | 142^tuv^ | 0.0130^fghi^ | 7 |
| 56 NBW Good | 122^jklmnopqrst^ | 0.0118^fghi^ | 6 |
| 56 NBW Poor | 120^kmnopqrstu^ | 0.0081^bdefgh^ | 7 |

**Supplementary Table S2.** The average number of observed OTUs and *Ruminococcaceae UCG-005* relative abundance for the interaction between days of age, birthweight class (BiW class; low (LBW) or normal (NBW)) and average daily gain class (ADG class; Good or Poor). Significant differences between interactions are identified by differences in the assigned Tukey HSD letters.
