# Supplementary material for: Changes in Faecal Microbiota Profiles Associated With Performance and Birthweight of Piglets
Source: Front Microbiol. 2020 Jun 11;11:917. doi: 10.3389/fmicb.2020.00917 (PMC7300224; doi:10.3389/fmicb.2020.00917)
Supplement: Supplementary file 3 [file Data_Sheet_1.DOCX]

**Supplementary material S1**

**Animal housing and management**

Gestating sows, managed in a 3-week batch farrowing system, were moved from a small-group straw yard system to a conventional part-slatted farrowing pen with crate at approximately 110 days of gestation, with gilts entering later at 113 days. Prior to entry, the farrowing pen was washed and disinfected (concentration = x 0.03 PhenoPharm, East Riding Farm Services, UK) and allowed to dry for a minimum of 7 days. Sows which had not farrowed by 116 days of gestation were induced with intramuscular injection of 2ml of a prostaglandin analogue (Planate, Intervet UK, Walton, UK). Sow diets were based on barley and soyabean and fed as a dry, home milled meal. The diet for gestating sows and gilts (13.14 MJ DE /kg, 13.82% CP and 0.62% lysine) was fed at 1.8kg/head/day throughout gestation and then at 1kg/head/day split over two feeds once housed in farrowing crates. Once all the sows in the batch had farrowed, sows were fed a lactation diet (13.98% MJ DE/kg, 18.50% CP and 0.95% lysine) initially as a 1kg/head/day allowance, which was increased to appetite by 0.5 kg/head/day until a 10 kg/head/day limit was reached. Water was available through a nipple drinker, *ad libitum*. The farrowing house was maintained at 21°C, whilst an enclosed heated creep area was available to piglets; this contained wood shavings as bedding for the first week of life. Water was available to piglets *ad libitum* through piglet nipple drinkers positioned at the rear of the sow. Piglets had their canine teeth clipped within 24 hours of life, and at 3 days of age were tail docked and received a 1ml intramuscular injection of iron (200mg/ml Ferroferon, Iron4you, Denmark). Piglets received the first of their 2-stage vaccination program against *Mycoplasma hyopneumoniae* (1ml M+PAC, Intervet, UK) at 7 days of age. Creep feed (16.50% DE MJ/kg, 22.50% CP and 1.7% lysine; FlatDeck 1, A-One Feeds Supplements Ltd, Thirsk, UK) was made available to experimental piglets from 10 days of age at a rate of 25g/pen/day, increasing up to 150g/pen/day during the final week of lactation. The creep feed was scattered in the creep area which had a solid floor.

At weaning (28 days of age ±1 day), experimental piglets were housed in fully slatted, temperature controlled flat deck accommodation. Room temperature was initially set to 26°C and reduced by 0.2°C/day to a minimum of 22°C, which was sustained for the remaining trial period. Experimental pigs were housed in pens of 20 piglets according to birthweight class, thus segregating sibling pairs of LBW and NBW piglets into pens of either LBW or NBW pigs only. Each pen was furnished with two nipple drinkers and two multi-space feed troughs. Piglets received a three-stage weaner starter diet regime (A-One Feeds Supplements Ltd, Thirsk, UK) for one week duration per diet to prevent differences in rates of diet consumption between LBW and NBW pigs resulting in different dietary substrates being consumed at fixed faecal sampling ages, thus avoiding diet composition effects on the microbiota from confounding differences related to BiW or ADG classes. At the point of weaning, pigs received their 2^nd^ stage vaccine against *Mycoplasma hyopneumoniae* in addition to a porcine circovirus type 2 vaccine, as intramuscular injections (1ml M-PAC, MSD Animal Health, Milton Keynes, UK; 1ml Ingelvac CircoFLEX; Boehringer Ingelheim, Duluth, USA).
