# Supplementary material for: Changes in Faecal Microbiota Profiles Associated With Performance and Birthweight of Piglets
Source: Front Microbiol. 2020 Jun 11;11:917. doi: 10.3389/fmicb.2020.00917 (PMC7300224; doi:10.3389/fmicb.2020.00917)
Supplement: Supplementary file 4 [file Data_Sheet_2.docx]

**Supplementary material S2**

**Analysis of sequenced data**

Processing of raw sequencing reads was performed in QIIME 2 (Bolyen *et al*., 2019, v 2018.8) (available at: <https://qiime2.org/>). Paired end sequencing reads were imported into QIIME 2 in the cassava 1.8 paired-end demultiplexed Fastq format, based on unique PCR index barcodes used for each sample. PCR primers were removed using the “cutadapt” plugin (Martin, 2011) and the *trim-paired* method. Paired end reads were joined using the plugin “vsearch” (Torbjørn *et al*., 2016) and command *join-pairs*, with a minimum of 35 mismatches in overlapping bases, minimum overlap of 210 base pairs and maximum merge length of paired end reads of 260 base pairs. A quality filter was applied to the merged reads using the “quality-filter” plugin (Bokulich *et al*., 2013). Based on the default setting, merged sequences were only retained when they had a PHRED score over a threshold value of 4. Sequences were truncated where more than 3 base calls in succession had a PHRED score below the threshold value. Resulting sequences were then only retained if, after truncation, the sequences were at least 75% the length of their original sequence. Quality of sequences were then visually assessed using the plugin “demux”. Merged sequences were denoised as an additional quality control step using the “deblur” plugin (Amir *et al*., 2017). Deblur utilizes a positive alignment-based filtering by specifying a reference database. Although the reference database was not used for taxonomic assignment at this stage, for the purpose of this study the SILVA 16s rRNA reference database (release version 132) was selected (Quast *et al*., 2013). The deblur steps output unique denoised sequences termed Amplicon Sequence Variants (ASVs); ASVs equate to OTUs and were referred to as this for all subsequent analysis stages. Trim length was set to 253, this resulted in an outputted OTU table and file of taxa associated with OTUs (taxonomy file). Chimeric sequences were detected using the “vsearch” plugin and the *uchime-denovo* method (Rognes *et al*., 2016). Once identified, chimeras were removed from the OTU table and taxonomy file using the “feature-table” plugin and the *filter-features* and *feature-seqs* commands, respectively. The OTU table was then assigned taxonomy using the SILVA database and the 16S rRNA V4 region to determine taxonomy (Quast *et al*., 2013), as performed by the “feature-classifier” plugin (Bokulich *et al*., 2018) and *classify-consensus-vsearch* method (Rognes *et al*., 2016). This method was selected over *classify-consensus-blast* as it searched the whole SILVA database for taxonomic assignments before selecting the most appropriate taxonomy, as opposed to the first positive match which may not always be the most accurate taxonomic assignment.

Archaea, mitochondria and chloroplasts were filtered from the OTU table and taxonomy file using the “taxa” plugin and the *filter-table* and *filter-seqs* commands respectively. The OTU table and taxonomy file were rarefied to 1000 reads per sample, to avoid sequencing depth bias in downstream analysis, whilst retaining as many samples in the analysis as possible at a viable sequencing depth to enable diversity to be captured. Rarefaction of the OTU table was performed by the “feature-table” plugin and *rarefy* command. Subsequently, the taxonomy file was filtered to only contain taxa retained after rarefaction by the *filter-seqs* command of the “feature-table” plugin. A phylogenetic tree was created by aligning sequences using the “alignment” plugin and the *mafft* method (Katoh and Standley, 2013), any unobserved or gapped regions of columns were then masked from the alignment using the *mask* method (Lane, 1991). A tree file was then generated using the “phylogeny” plugin and the *FastTree* method to create the phylogenetic tree (Price, Dehal and Darkin, 2010). The tree was then rooted so that a UniFrac distance matrix could be calculated in downstream analysis using the *midpoint-root* method, enabling the origin of phylogeny to be determined. QIIME 2 does not generate OTU tables that contain taxonomy information as in QIIME 1. For downstream analysis, taxonomic information as part of the OTU table is required and was added to the OTU table using the BIOM format software (McDonald *et al.*, 2012), and the *add-metadata* command. The OTU table was then converted to tab-separated value (tsv) format to enable statistical analysis of taxa abundance to be conducted, using the *convert* command in BIOM format. The additional options *--header-key taxon* and *--tsv-metadata-formatter naïve* were also included in the command.

For the statistical analysis of the data the dependant variables consisted of piglet liveweight at each sampling age, observed OTUs and Shannon alpha diversity indices, Bray Curtis beta diversity distance matrix and the proportional abundance of the 20 most abundant taxa. Each model was built manually in a forwards-backwards stepwise fashion, with any non-significant fixed effects or interactions removed from the final model. All statistical models were fitted using the maximum likelihood method.

The glmmTMB models utilised to analyse associations within the 20 most abundance genera and the fixed effects of interest failed to converge for three taxa, so the models were consequently fitted with piglet ID as a fixed variable within the model using the “betareg” package (Cribari-Neto and Zeileis, 2010, v 3.1-2). The validity of beta regression models fitted using *betareg* was determined through inspection of a half-normal plot of standardised residuals and a scatter graph of standardised residuals plotted against fitted values. The *Bacteroides* and *Prevotella* 1 models did not display major deviations from the assumed normality or homoscedasticity assumptions of the beta regression model. However, the *Escherichia-Shigella* model violated these assumptions. Based on the Cook’s Distance plot, 12 samples were removed from the dataset as these samples corresponded to a Cooks Distance of over 0.07 and to samples identified as having 0 abundance or being outliers in the dataset.

**References**^†^

Bokulich, N. A., Kaehler, B. D., Rideout, J. R., Dillon, M., Bolyen, E., Knight, R., Huttley, G. A., and Caporaso, G. J. (2018). Optimizing Taxonomic Classification of Marker-Gene Amplicon Sequences with QIIME 2’s Q2-Feature-Classifier Plugin. *Microbiome.* 6 (1), 90. doi: 10.1186/s40168-018-0470-z.

Bokulich, N. A., Subramanian, S., Faith, J. J., Gevers, D., Gordon, J. I., Knight, R., Mills, D. A., and Caporaso, J. G. (2013). Quality-Filtering Vastly Improves Diversity Estimates from Illumina Amplicon Sequencing. *Nat. Methods.* 10 (1), 57-59. doi: 10.1038/nmeth.2276.

Katoh, K., and Standley, D. M. (2013). Mafft Multiple Sequence Alignment Software Version 7: Improvements in Performance and Usability. *Mol. Biol. Evol.* 30 (4), 772-80. doi: 10.1093/molbev/mst010.

Lane, D, L. “16s/23s rRNA sequencing”. In: Stackebrandt, E. and Goodfellow, editors. Nucleic Acid Techniques in Bacterial Systematics. New York: John Wiley and Sons (1991). p. 115-175.

McDonald, D., Clemente, J. C., Kuczynski, J., Rideout, J. R., Stombaugh, J., Wendel, D., et al. (2012). The Biological Observation Matrix (Biom) Format Or: How I Learned to Stop Worrying and Love the Ome-Ome. *GigaScience* 1 (1), 7. doi: 10.1186/2047-217X-1-7.

Price, M. N., Dehal, P. S. and Arkin, A. P. (2010). FastTree 2 – Approximately Maximum-Likelihood Trees for Large Alignments. *PLOS ONE* 5(3), e9490. Doi: 10.1371/journal.pone.0009490

^†^ References not included in the main text of the manuscript; all other citations are referenced in the main text.
